# Analyzing the factors influencing adoption intention of internet banking: Applying DEMATEL-ANP-SEM approach

**DOI:** 10.1371/journal.pone.0227852

**Published:** 2020-02-05

**Authors:** Wan-Rung Lin, Yi-Hsien Wang, Yi-Min Hung

**Affiliations:** Department of Banking & Finance, Chinese Culture University, Taipei, Taiwan; The Bucharest University of Economic Studies, ROMANIA

## Abstract

The main purpose of this study is to propose a research model to explore the key factors affecting consumers’ willingness to use online banking. There are two stages in this research. Firstly, the decision making trial and evaluation laboratory (DEMATEL) and analytic network process (ANP) were used to explore the key factors of companies in operation of online banking. Secondly, the structural equation modeling (SEM) was used to explore the key factors of consumers’ actual use of online banking. The results showed differences in the factors that companies and consumers adopted. Based on the findings, companies can adjust their business strategies and improve the consumers’ willingness of online banking usage. The primary factor valued by both companies and consumers is trust. Hence, in the business of internet banking, the companies must strengthen areas such as liquidity monitoring, information security, and compliance with financial regulations, in order to reduce risks and gain customers’ trust.

## I. Introduction

With technological innovation and changes in life styles, business interactions between consumers and banks are gradually changing from the handling of business by customers in person in physical banks to having access to the financial services they need directly via technologies such as Internet banking or mobile banking [1, 2]. [3] stated that the future development trend of banks will be that the “bank will not only be a place, but a kind of behavior”. That is to say, the business model in which a banking industry operator provides services to consumers through a physical bank will be phased out, while consumers’ needs for financial services will be satisfied via digital, Internet-based, and mobile financial services.

In recent years, financial technology has set off a huge wave all over the world and such disruptive innovation that combines technology with financial services has posed a threat to the business model of the traditional financial sector [4]. Amid this wave, the banking industry was the first to bear the brunt. Fintech could reduce the dependence on traditional financial institutions and provide low-cost and higher-value financial services. However, it will incur the issues that are most concerned by consumers, including privacy, information security, and consumer protection, if the involvement and supervision of financial institutions is inadequate [5, 6, 7]. Overall, the rise of Fintech has brought about a great impact on the banking industry, which has changed the banking ecosystem and even subverted previous business models and service modes [8]. In view of the above, in order to follow the future trend and satisfy consumers’ requirements, banking operators must make adjustments and improvements to existing financial services.

Internet banking is a financial service that has been developed by banking operators for many years, and which provides real-time, fast and convenient services. Consumers can have access to various banking services such as online transfers, payment of bills, currency exchange services, enquiries of account data, and financial investments after applying for a set of accounts and passwords of their own [9, 10]. Thanks to Internet banking, consumers can significantly reduce the waiting time required for over-the-counter service and can be free from the limitations of the business hours of the bank.

With the development of Internet banking, consumers have already gotten used to the services it provides. In addition, Internet banking provides a greater security due to highly protected account privacy in the system and well-developed dispute settlement mechanisms, claim laws and regulations, as compared with early forms of Fintech. However, in the wave of Fintech, the inherent system defects of Internet banking will hinder its development, such as restricted amounts of online remittance and incompatibility with the system of the device used by users. Based on the above, this study aimed to identify the key factors influencing the use of Internet banking. Discussions were conducted from the aspect of business operator and consumer use, respectively. The degree of recognition of such factors by experts and consumers was integrated through expert questionnaires and consumer questionnaires, and the differences and similarities between the two were also analyzed. The research results could be used as a basis for banking operators to develop strategies for improving Internet banking, as well as a reference indicator for consumers in using Internet banking.

Nowadays, consumers can effortlessly search and compare the websites of banks in terms of the quality and content of the services provided. Compared with physical bank branches, the rapid growth of Internet banking could bring banking industry operators great benefits, as well as fierce horizontal competition. For banking industry operators, it is of great importance to know expert business strategies for Internet banking and the basis for consumers to use Internet banking. Accordingly, this study aimed to address the following research problems:

1. In operating Internet banking, banking industry operators should give priority to the influence factors that will improve consumer’s intention to use Internet banking.
2. In using Internet banking, which influence factor will be prioritized by consumers to decide whether or not to continue to use the Internet banking?
3. What are the differences in the influence factors considered by banking industry operators and consumers?

This study engaged in an in-depth discussion on five factors that influence consumer use of Internet banking services, namely, perceived usefulness, perceived ease of use, perceived risk, trust, and satisfaction, through expert questionnaires and consumer questionnaires. Accordingly, research purposes of this study were as follows:

1. To discuss the factors influencing the business strategy for Internet banking through expert questionnaires based on ANP.
2. To discuss the factors influencing consumer use of Internet banking through consumer questionnaires based on SEM.
3. To discuss the differences in the influence factors considered by banking operators and consumers by integrating the results of the expert questionnaires and consumer questionnaires.

## II. Literature review

### 1. Internet banking

Electronic banking started to rise in 1960 and was mainly represented by Automated Teller Machines (ATM) as an extension of over-the-counter service, followed by the new financial services derived from the development of computer technology. For the main purpose of electronic banking, consumers could conduct business interactions with banks via communication devices and could have access to services without going to bank branches. The operation of electronic banking been focused on banking services until the 1990s. Except for certain services that could be completed via an ATM, consumers were still required to go to bank branches to handle most business. Over recent years, the well-developed Internet technology has brought the rise of a brand-new type of financial service system, namely, Internet banking. The first security first network bank (SFNB) in the world started to operate online in 1995.

Internet banking enables customers to have access to remote financial services, such as enquiries of account data, payment of bills, financial payments, and online transfers, not in bank branches, but through the Internet via information devices such as PCs, Notebook PCs, and mobile devices [11]. With Internet banking, there is neither a physical branch nor counter staff, and consumers can have access to the transactions or services they need anytime and anywhere [12, 13]. By providing Internet banking services, bank operators can benefit from many advantages and improve corporate competition, such as reducing the time to handle transactions as well as the cost and labor involved in establishing branches, so as to improve service quality and customer satisfaction.

Consumers can use Internet banking without purchasing any software device and they do not need to store or backup data, as all transaction information is stored at the bank terminal. However, such transaction information also includes the private information of consumers. In case of malicious attacks on Internet banking or bank staff misconduct, damages will be caused to the rights and interests of the consumers, and consumer use of Internet banking will be further affected. Therefore, how to operate Internet banking to provide consumers with a safe and convenient environment and how to improve consumer trust and satisfaction with Internet banking are topics that banking operators must consider.

### 2. Perceived usefulness and perceived ease of use

**Fig 1:**
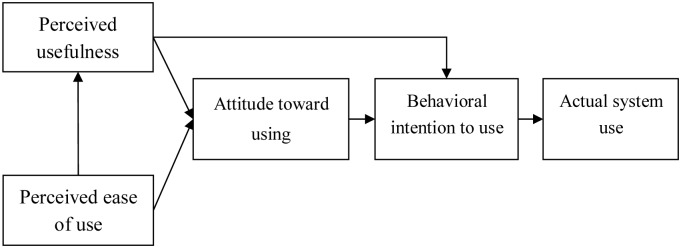
Technology acceptance model.

[14] proposed the Technology Acceptance Model (TAM) to analyze why enterprises or individuals will use a new technology. In such a model, perceived usefulness is defined as the degree to which a user believes that using a new technology will improve his working performance and perceived ease of use is defined as the degree to which a user believes that using a new technology will be effortless. Perceived usefulness and perceived ease of use influence attitude toward using, further influence behavioral intention to use, and ultimately influence actual system use, as shown in Fig 1. The relationship between attitude toward using and behavioral intention to use represents that a user will generate behavioral intention to use for the execution of a certain behavior when he/she has a positive attitude toward the result of the certain behavior.

In most prior studies discussing Internet banking, perceived usefulness and perceived ease of use were included as factors to influencing consumer use of Internet banking. [15] believed that a consumer will be more willing to use Internet banking services when there is higher perceived usefulness and perceived ease of use for Internet banking. In other words, banking industry operators can improve consumer use intention if Internet banking is designed to be easy to operate and can improve consumer’s transaction efficiency [16, 17].

In TAM, perceived usefulness and perceived ease of use can not only influence consumer use intention but also influence other factors [18]. [19] found that consumers will have a higher perceived usefulness for Internet banking in the case of a higher perceived ease of use for Internet banking. [20] also found that consumers will be more satisfied with the services provided by Internet banking in the case of a higher perceived usefulness for Internet banking [21].

According to the above literature review, perceived usefulness and perceived ease of use were included in the research dimensions of this study, and perceived usefulness was defined as the degree to which a consumer believes that in using a new system, the information provided by such system will improve transaction efficiency, which indicates that a consumer expects to improve his/her transaction efficiency in using Internet banking. Meanwhile, perceived ease of use was defined as the degree to which a consumer believes that using new technology would be effortless, which indicates that consumers will effortlessly complete banking business in learning or operating a system interface.

### 3. Perceived risk

For quite some time, researchers have considered perceived risk as an important factor influencing consumer behavior in transactions and have verified its influence on consumer consumption decisions. Research has found that consumers will choose not to conduct a certain transaction if they have a higher perceived risk for the transaction [22, 23, 24]. In prior studies, perceived risk was defined as a consumer’s personal subjective feeling of potential loss in developing consumption decisions or purchasing a product or service [25, 26].

Consumers need to provide their private information before getting access to the online services provided by a company when they conduct transactions via telephone or the Internet; therefore, data security issues will be incurred. Data security issues, in turn, will increase consumers’ perceived risk for transactions via telephone or Network, which will further influence consumer use of online transactions [27, 28, 29]. Data security refers consumers delivering private financial information via the Internet so as to comply with the regulations of a company, which company staff or other third parties may steal and use without authorization [30, 31].

In prior studies discussing Internet banking, perceived risk was included in the factors influencing consumer use of Internet banking. Those studies found that consumers will choose not to use Internet banking in the case of a higher perceived risk [32, 33, 34]. Therefore, in this study, perceived risk was regarded as one of the research dimensions and was defined as the risk perceived by consumers in using Internet banking services, such as transaction interruptions due to network instability, transaction amount errors, and leakage of personal information.

### 4. Trust

Prior scholars believed that the e-commerce environment involves more uncertainties and risks. As a result, in the development of e-commerce, trust is a critical factor [35, 36, 37, 24]. As both parties in a transaction are located in different places and the Internet banking account used by a consumer can only be protected by the security strategy and information system provided by the banking operator to a great extent, consumers will be exposed to the risk of uncertainties in using Internet banking accounts [38].

The nature of transactions conducted via Internet banking is different from those conducted via a physical bank. Users must deliver important files or private information via the Internet, and damage will be caused to the rights and interests of users in the case of security flaws. As a result, users will not trust Internet banking and even refuse to use Internet banking services [39, 40, 41, 42]. In providing Internet banking services, banking operators must try to reduce user concerns about the security of Internet banking, build user faith and trust, and further improve their intention to use Internet banking [43, 13, 44, 45].

In most prior studies discussing Internet banking, trust was included in the factors influencing consumer use of Internet banking. [46] believed that consumers will be more willing to use Internet banking services if they have higher trust with Internet banking. In other words, banking operators can improve consumer use intention if they can make consumers believe that it will be safe to use Internet banking [47, 48].

Trust will not only influence consumer intention to use Internet banking services but also influence other factors. Consumers will have higher perceived usefulness and satisfaction with Internet banking if they have higher trust in Internet banking [49].

According to the above literature review, trust was regarded as one of the research dimensions of this study and was defined as consumers trusting the reliability and integrity of Internet banking [50, 51, 52].

### 5. Satisfaction

In recent years, satisfaction has been widely discussed in studies in the field of information systems and e-commerce and is considered as the key factor influencing customer retention and consumer loyalty [53].

[54] proposed the expectancy-disconfirmation theory to explain customer satisfaction. Consumers will generate expectations for the performance of a product or service before buying and will generate perceived performance after obtaining the product or service. Consumers will determine their satisfaction by comparing the difference between expectation and perceived performance. There will be a positive disconfirmation phenomenon if the perceived performance is greater than the expectation, which will improve customer satisfaction; however, there will be a negative disconfirmation phenomenon if the perceived performance is less than the expectation, which will reduce customer satisfaction. Prior studies have found that users will continue to use a product or service if they are more satisfied with such information regarding a product or service. That is to say, there is a positive correlation between satisfaction and willingness for continuity [55, 56].

In prior studies discussing Internet banking, satisfaction was included in the factors influencing consumer use of Internet banking, and such studies found that consumers will be more willing to use Internet banking services if they are more satisfied with Internet banking [57, 20]. Accordingly, satisfaction was regarded as one of the research dimensions of this study and was defined as the degree of consumer feelings about Internet banking services after use.

## III. Research method

### 1. Research subjects

#### (1) Business operators (DEMATEL-ANP)

In this research, the questionnaire survey was conducted in two stages. DEMATEL questionnaire was used in the first stage, and ANP questionnaire was used in the second stage with the respondents same as the experts in the first stage. This research explored the keys factors of online banking usage, and its influence factors were multi-criteria, with feedback and interaction among all criteria. Hence, this research used ANP, which could solve the above problems [58]. ANP was used to establish the correlation among the factors of online banking usage, and determine the weights of all factors. In terms of expert selection, due to the limited application rules of ANP, the number of experts should not be large and 5–15 persons [58, 59, 60, 61, 62]. A large number of interviewed experts or deviated selection criteria would affect the consistency of analysis results, which was difficult to conform to the actual situations. Hence, seven supervisors and scholars who meet the criteria of “having engaged in the banking industry or research in related fields for more than 5 years and having basic knowledge, operational skills and experience in online banking” were selected in this research. They are bank industries (two experts), financial scholars (three experts), financial company consultants (two experts).

#### (2) Consumers (SEM)

The research hypothesis was established based on the analysis results of DEMATEL, and SEM was used for further analysis. In this part, a questionnaire survey was conducted on consumers with a 5-point [63], and distributed online to the consumers who have actually used online banking. A total of 398 questionnaires were collected, including 373 valid questionnaires, with an effective recovery rate of 93%. [64] indicated that the number of samples should be more than 5 times of the questionnaire items. There were a total of 23 items in the SEM questionnaire of this research, thus, at least 115 samples were required. A total of 373 samples were collected in this research, so the analysis results had high reference value.

### 2. DEMATEL

#### (1) Origin and purpose of DEMATEL

The DEMATEL technique was proposed by the Battelle Memorial Institute in Geneva in 1973 [65] and was originally used for research on intricate and difficult problems for the purpose of assisting in collecting problems and obtaining integrated solutions and hence getting a better understanding of problem related groups. This technique can be used to effectively identify intricate causal relationship structures, calculate the casual relationships among all elements via the degree of influence between two factors by applying a matrix and related mathematical theory, and illustrate the degree of casual influence in figures. The technique is widely applied in user investment strategies in mobile transaction systems, corporate governance, and urban planning, etc. [66, 67, 68].

In recent years, DEMATEL has been applied in more studies analyzing the casual relationship and degree of influence between factors. As a result, in this study, before the application of this technique, the relationship between factors influencing the use of Internet banking was tested; then, results were imported into ANP and SEM, and ultimately the priority of such factors was identified.

#### (2) Execution steps of the DEMATEL [69]

*1*. *Define factors and identify scales*. In this step, definitions of the factors were listed through a literature review and brainstorming, etc. The questionnaires were then designed. Finally, the degree of influence between two factors was judged according to an expert subjunctive mental model. In this study, five scales were used for representing the degree of influence between factors, in which 0 = no influence, 1 = low influence, 2 = medium influence, 3 = high influence and 4 = very high influence.

*2*. *Find the direct-relation matrix*. The number of criteria was *n*. Based on the experts’ opinions obtained through questionnaires, pairwise comparisons of the criteria in respect of the influence relationship and degree of influence were conducted, and an *n* × *n* matrix was obtained. *H* illustrates the direct-relation matrix, as shown in Eq 3-1:

$$H=\left[h_{ij}\right]=\left[\begin{array}{cccc} 0 & h_{12} & \cdots & h_{1n}\\ h_{21} & 0 & \cdots & h_{2n}\\ \vdots & \vdots & \ddots & \vdots \\ h_{n1} & h_{n2} & \cdots & 0 \end{array}\right],i=1,2,\ldots ,n;\;j=1,2,\ldots ,n$$ (3-1)

*3*. *Normalize the direct-relation matrix*. Define $\beta =\frac{1}{{\text{max}}_{0\leq i\leq 1}(\sum_{j=1}^{n}h_{ij})}$, and multiply matrix element by *β*; *X* illustrates the normalized direct-relation matrix, as shown in Eq 3-2:

$$X=h_{ij}\times \beta ,i=1,2,\ldots ,n;j=1,2,\ldots ,n$$ (3-2)

*4*. *Calculate the total-relation matrix*. Define lim_*k*→∞_ *X*^*k*^ = 0, the total-relation matrix *T* is as shown in Eq 3-3, where, *I* illustrates a unit matrix:

$$T={\text{lim}}_{k\to \infty }(X^{1}+X^{2}+\ldots +X^{k})=X{\left(I-X\right)}^{-1}$$ (3-3)

*5*. *Produce a causal diagram*. Define *T* = [*t*_*ij*_]_*n*×*n*_, (*i*, *j* = 1, 2, …, *n*)as all elements in *T*, and define *D* and *R* as the vector of *n* × 1 and represent the sum of the columns and sum of the rows in relation matrix *T*, as shown in Eqs 3-4 and 3-5:

$$D={\left[D_{i}\right]}_{n\times 1}=\sum_{i=1}^{n}t_{ij},i=1,2,\ldots ,n$$ (3-4)

$$R={\left[R_{j}\right]}_{n\times 1}=\sum_{j=1}^{n}t_{ij},j=1,2,\ldots ,n$$ (3-5)

*D*_*i*_ demonstrates the total influence of element *i* as the cause on other elements, and *R*_*j*_ demonstrates the total influence of other elements on element *j* as the effect. The value of (*D*+*R*) demonstrates the total degree of influence of and on the element, by which the prominence of the element in the problem group is obtained. (*D*-*R*) demonstrates relation; a positive value represents that the element is a dominant element while a negative value represents that the element is an influence element. (*D+R*) and (*D-R*) represent the horizontal axis and vertical axis of the causal diagram, respectively. As independence among factors is considered in the DEMATEL technique, it is more suitable for research on problems in the real world, as compared with traditional research method. By calculating and applying the DEMATEL technique, the degree of correlation among factors and the dependence and feedback of factors in the network architecture could be identified [70, 71]. Then, results in this study were imported into the ANP architecture to form DANP (DEMATEL-based ANP) and calculate the weights of factors [72, 73]. In addition, the architecture in the aspect of consumer use was established and research hypotheses were proposed based on the results.

### 3. Analytic network process (ANP)

In recent years, the Analytic Network Process (ANP) has been widely applied in decision problems under uncertainties and multiple criteria. It is mainly used to divide a decision problem into a vertical hierarchy architecture. It only considers the influence between upper and lower hierarchies, and it assumes that elements in the hierarchies are independent of each other. However, in measuring various decision problems in real life, not only the elements in the same hierarchy will influence each other, but also elements in different hierarchies will influence each other, illustrating not only a linear hierarchy-upon-hierarchy relationship but a network-like relationship. Therefore, [58] proposed the ANP technique, which takes interdependence and feedback among criteria and alternatives into account to overcome the issue that the traditional AHP technique cannot address intricate decisions.

In practice, the ANP technique provides decision makers with a set of evaluation scales, which includes five items, namely, 1 = equal importance, 3 = weak importance, 5 = considerable importance, 7 = great importance and 9 = absolute importance, and four items between these five scales: 2, 4, 6, and 8 [59]. Decision makers should input the measurement criteria judged by them in the questionnaire based on their basic knowledge and experience about the topic and then calculate the weight of each factor in the whole decision architecture using the values obtained, and finally identify the key influence factors [74].

In prior studies, the ANP process was divided into six steps [58, 59, 75], as explained below.

#### (1) Find the pairwise comparison matrix

In this step, ANP questionnaires are converted into a matrix and the pairwise comparison matrix *H* is calculated in Eq 3-6:

$$H={\left[h_{ij}\right]}_{n\times n}\left[\begin{array}{cccc} 1 & h_{12} & \cdots & h_{1n}\\ h_{21} & 1 & \cdots & h_{2n}\\ \vdots & \vdots & \ddots & \vdots \\ h_{n1} & h_{n2} & \cdots & 1 \end{array}\right]$$ (3-6)

#### (2) Expert preference integration

As there are several decision makers and each respondent may have different cognition of questions, pairwise comparison values may vary. As a result, in this study, pairwise comparison values of all experts were integrated into a pairwise comparison matrix by the geometric mean.

#### (3) Calculate eigenvalues and eigenvectors

Upon completion of integration of the pairwise comparison matrix, eigenvector *W*_*ij*_ is obtained in Eq 3-7, where, *λ*_max_ represents the maximum eigenvalue in matrix *H*:

$$HW_{ij}=\lambda_{man}W_{ij}$$ (3-7)

#### (4) Consistency test

Upon completion of establishing the pairwise comparison matrix, a consistency test must be conducted, in order to confirm the consistency among the judgment of the respondents. *λ*_max_ is obtained by following the above steps and the consistency index (CI) is calculated, as shown in Eq 3-8:

$$CI=\frac{\lambda_{\text{max}}-n}{n-1}$$ (3-8)

Then, a random index is calculated according to the order of the pairwise comparison matrix and its values are as listed in Table 1. Upon obtaining CIs and RIs, the consistency ratio (CR) is obtained, as shown in Eq 3-9.

**Table 1:** Comparison table of RIs.

Order of the matrix	1	2	3	4	5	6	7	8	9	10
RI	0	0	0.52	0.89	1.11	1.25	1.35	1.40	1.45	1.49

$$CR=\frac{CI}{RI}$$ (3-9)

[59] stated that *CR* ≤ 0.1 demonstrates that the degree of difference in judgment on the weights of factors among decision makers is within an acceptable range, i.e., there is consistency; *CR>0*.*1* requires a review of the questions and modifying the judgment on pairwise comparisons.

#### (5) Find the supermatrix

In order to evaluate the dependence between criteria, a supermatrix is established. Each evaluation scale in the matrix represents the influence of the elements within a criterion on the elements in other criterion (external dependence) as well as the influence on the elements within the same criterion (interdependence); however, not all elements will influence other elements. In such a case, *w* represents the relationship between two elements. Finally, the elements in all criteria are listed on the left and top of the matrix, respectively, to form a complete integrated matrix called a supermatrix.

#### (6) Operation of the supermatrix

Eigenvectors of the performance indicators of all criteria are integrated into a large matrix which is called an unweighted supermatrix. The unweighted supermatrix is multiplied by the eigenvectors obtained by the pairwise comparison matrix of the evaluation criteria to form the weighted supermatrix, which is represented by *W*. If the performance indicators depend on each other, a fixed convergence extreme will be obtained by multiplying matrix *W* several times, and the extreme will remain unchanged.

### 4. Structural equation modeling (SEM)

#### (1) SEM

SEM is a regression-based multi-variable technique that integrates two statistical approaches, namely, factor analysis and path analysis. It is a kind of data analysis method for confirmatory empirical study and can be used to discuss the relationship between multiple sets of variables for the purpose of analyzing the casual relationship among multiple variables to verify theory [76]. In applying such confirmatory study techniques, researchers must establish a research model based on theory; in other words, researchers can propose certain restrictions on the analytical model based on a theory or hypothesis.

[77] proposed the steps for establishing SEM. First, the theoretical model should be developed; then, a path diagram of the casual relationships among variables should be identified and parameters should be estimated. The goodness of fit of the model should be tested next; finally, revisions should be made to the model.

Generally, SEM is used to establish a theoretical basis through a literature review. In this study, however, discussion was conducted in view of practice. Casual relationships among variables were identified through DEMATEL questionnaires. As SEM has the apriority of theory, integrates statistical approaches, can measure and analyze problems at the same time, and puts emphasis on multiple indicators, it is applicable to processing the relationships among multiple variables. SEM currently is widely applied in researches on business strategy, consumer use intention, and other problems [78, 79].

In this study, the casual relationships among five dimensions, including perceived usefulness, perceived ease of use, perceived risk, trust and satisfaction, were discussed by applying the linear structure relationship (LISREL), and the goodness of fit, reliability, and validity of the model were tested to verify the model. Related measurement indexes were as listed in Table 2.

**Table 2:** Measurement indexes for goodness of fit of SEM.

Type	Name of index	Range	Acceptable value	Critical value
Overall Fit Index	Chi-Square value	--	The smaller, the better	
	Chi-Square value/ DOF	--	<5	<3
	RMSEA	0–1	<0.1	<0.05
	RMR	0–1	<0.1	<0.05
	SRMR	0–1	<0.1	<0.05
Comparative Fit Index	NFI	0–1	>0.9	>0.95
	NNFI	0–1	>0.9	>0.95
	IFI	0–1	>0.9	>0.95
	CFI	0–1	>0.9	>0.95
	RFI	0–1	>0.9	>0.95
Parsimonious Fit Index	PGFI	0–1	>0.5	Close to 1

### 5. Integration DEMATEL-ANP-SEM approach

Analytic Hierarchy Process (AHP) is proposed by [80]. The main difference between AHP and ANP is that, all criteria are deemed as independent groups in AHP, while there are interdependence and feedback among all criteria in ANP. As it is difficult to express the business models of online banking with general equations, quantitative models are needed to convert the model into quantity and graphs. DEMATEL and ANP are the quantitative approaches to solve complex system problems. With the characteristics such as theoretical apriority, integration of statistical approaches, simultaneous measurement processing and problem analysis, and attaching importance to multiple indicators, SEM is suitable for processing the relationship among multiple variables. Because there were strong feedback and interdependence among the key factors of consumers’ usage of online banking, so ANP was used to solve the complex relationship among all factors to reach an optimal solution. In addition, different from the traditional SEM, the purpose of combining DEMATEL and SEM in this research is to make the companies’ business strategies better meeting the consumers’ needs, so as to improve the usage rate of online banking.

**Fig 2:**
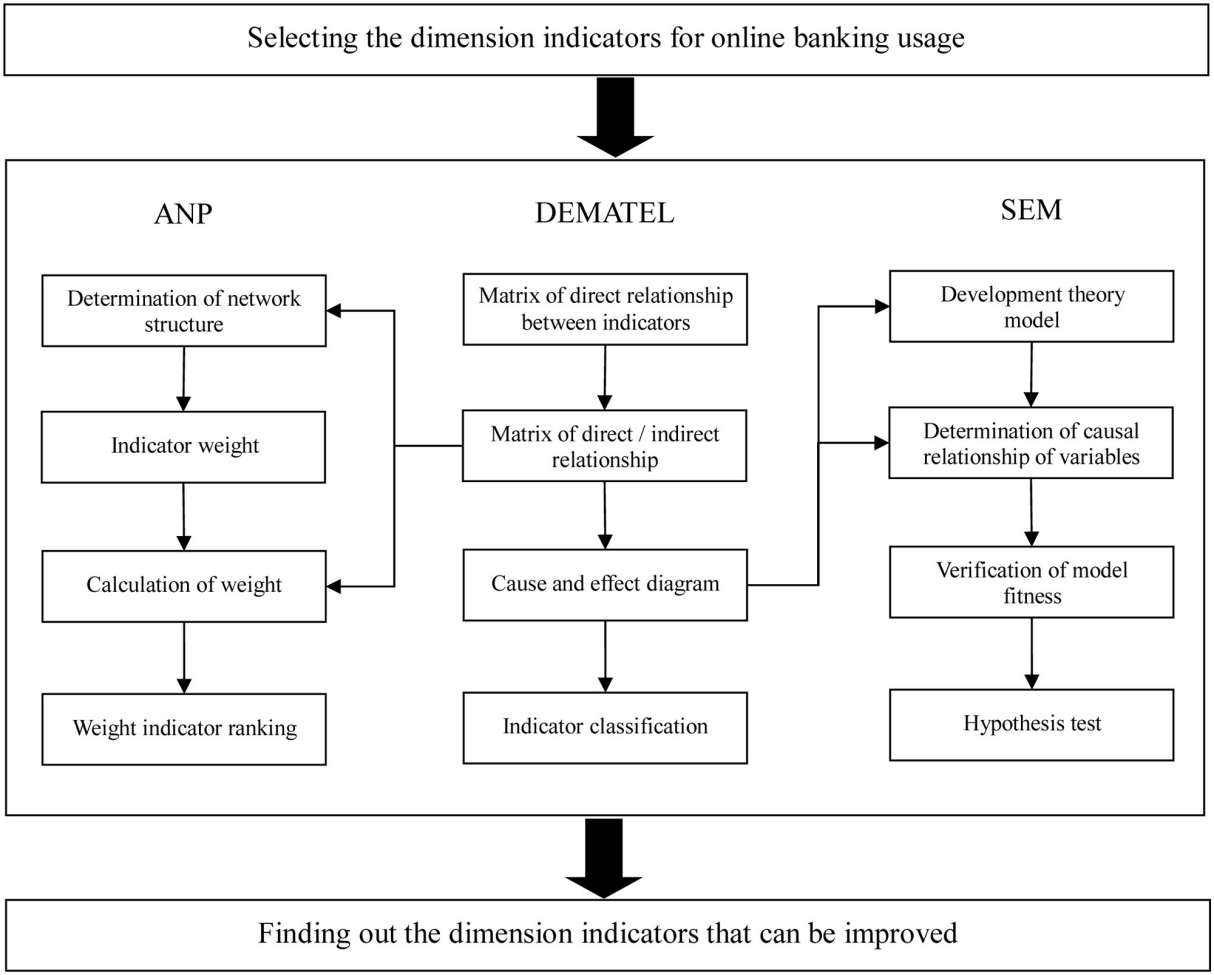
Applications of DEMATEL-ANP-SEM.

The research model flow established in this research is shown in Fig 2. Firstly, after selecting the dimension indicators for online banking usage, DEMATEL was used to determine the influence degrees and directions of all indicators, as well as the network structures of all indicators. Then ANP was applied to determine the relative weights of all indicators. Finally, the indicators were ranked and classified based on their properties by combining correlation degrees, weights and cause and effect diagrams, to find out the dimension indicators that can be improved. In addition, SEM was used to analyze the causal relationships among dimensions to find out the appropriate measuring indicators.

## IV. Empirical analysis

Three types of statistical software, including Super Decision, SPSS 22.0, and LISREL 8.80, were applied in this study for analysis, and suggestions were proposed based on the research results.

### 1. DEMATEL results

In this study, correlations among five dimensions, namely, perceived usefulness, perceived ease of use, perceived risk, trust and satisfaction, were measured by the DEMATEL technique to obtain the structural relation diagram of such dimensions. The diagram was used as a reference basis for designing ANP and SEM questionnaires. Upon recovery of the questionnaires in Stage 1, the total-relation matrix was calculated in Eq 3-3, as listed in Table 3. Then, the sum of the columns (D), the sum of the rows (R), and the combined sum of the columns and rows (D+R) of the total-relation matrix were obtained in Eqs 3-4 and 3-5, as listed in Table 4.

**Table 3:** Total-relation matrix.

	Perceived usefulness	Perceived ease of use	Perceived risk	Trust	Satisfaction
Perceived usefulness	0.311	0.447	0.379	0.604	0.698
Perceived ease of use	0.471	0.308	0.413	0.592	0.702
Perceived risk	0.455	0.421	0.294	0.640	0.726
Trust	0.482	0.428	0.459	0.374	0.435
Satisfaction	0.387	0.409	0.361	0.562	0.395

**Table 4:** Comparison table of total-relation matrix.

	Sum of columns (D)	Sum of rows (R)	Combined sum of rows and columns (D+R)	Difference between the sums of rows and columns (D-R)
Perceived usefulness	2.439	2.106	4.545	0.333
Perceived ease of use	2.486	2.013	4.499	0.473
Perceived risk	2.536	1.906	4.442	0.630
Trust	2.178	2.772	4.950	-0.594
Satisfaction	2.114	2.956	5.070	-0.842

As shown in Table 4, the vector of the combined sum of the rows and columns for the dimension of satisfaction was the highest (D+R = 5.070), which demonstrated that the correlation between the dimension of satisfaction and other dimensions was the most significant. The difference between the sums of the rows and columns (D-R) represented the net relation in the total-relation matrix. Among the five influence dimensions, (D-R) of the dimensions for perceived usefulness (0.333), perceived ease of use (0.473), and perceived risk (0.630) were greater than 0, which demonstrated that the degree of influence of these three dimensions on other dimensions was greater than the degree of influence of the other dimensions. As a result, perceived usefulness, perceived ease of use, and perceived risk were influence dimensions; on the contrary, (D-R) for the dimensions of trust (-0.594) and satisfaction (-0.842) were less than 0, which demonstrated that the degree of influence of these two dimensions on other dimensions was less than the degree of influence of other dimensions. As a result, trust and satisfaction were the dimensions under influence.

**Fig 3:**
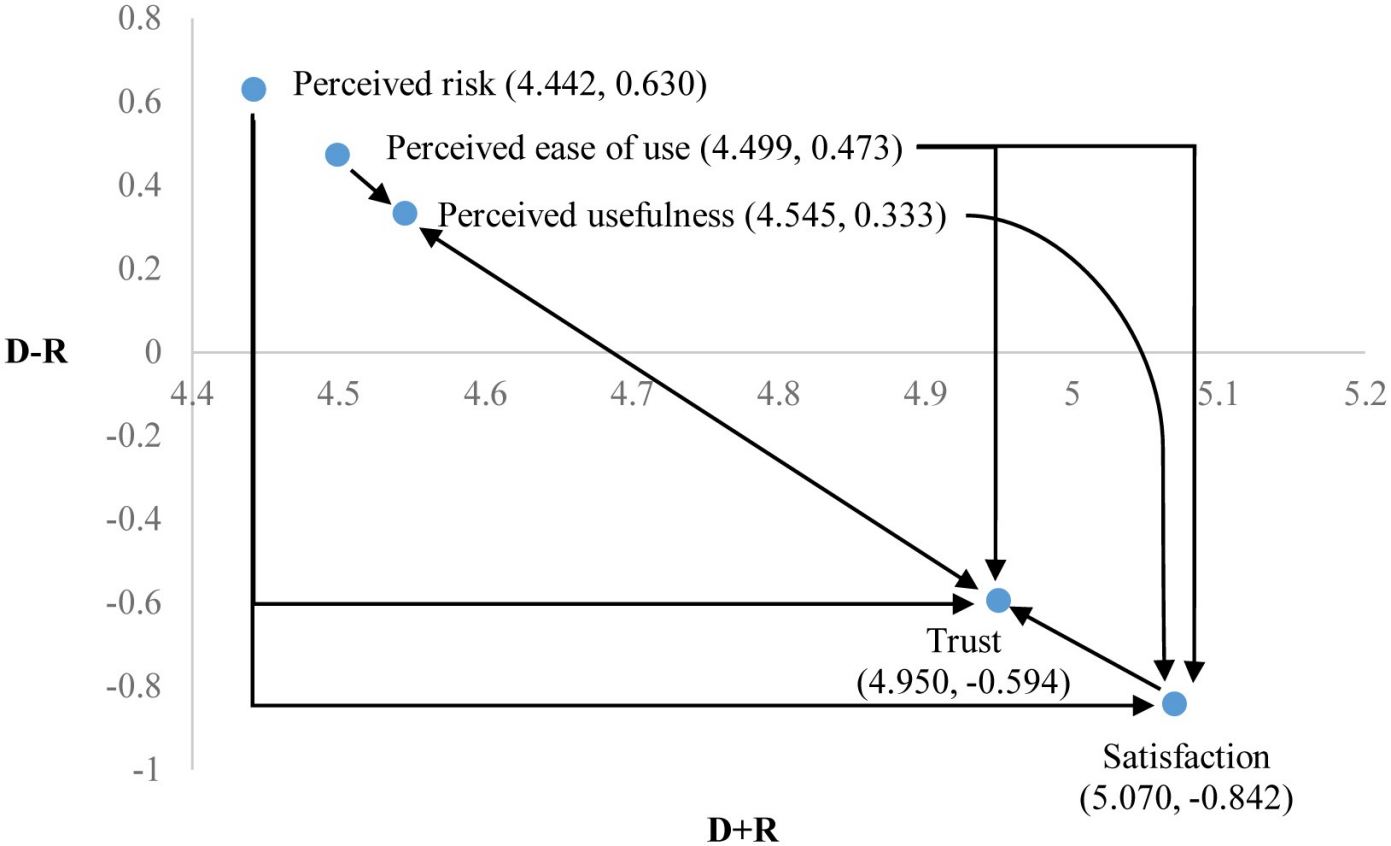
Structural relation diagram of dimensions.

In this study, the arithmetic average (0.470) was calculated according to the values listed in Table 3, and this value was used as the threshold value for the judgment of the correlations among dimensions. The correlations among dimensions could be judged based on the threshold value of 0.470. Further, a diagram was produced using (D-R) and (D+R) in Table 4, i.e., the structural relation diagram of the dimensions, as shown in Fig 3.

### 2. ANP results

The influence relationships among dimensions identified by the DEMATEL technique are shown in Fig 3. In this study, a questionnaire for Stage 2 was further designed. Interdependence and feedback among dimensions were addressed by the ANP technique and the weights of influence among dimensions were calculated; finally, the key influence dimensions were identified. ANP questionnaires were also dispatched to the seven experts who completed the DEMATEL questionnaires. These experts conducted pairwise comparisons of the degree of importance of the dimensions presented in the questionnaire using the 1–9 evaluation scales proposed by [58]. Upon recovery of the questionnaires, expert preference integration was conducted according to the geometric mean; then, Super Decision system software was applied to calculate the weights of the dimensions and the consistency among the experts’ evaluation was verified by a consistency test.

The analysis results found by the Super Decision software are listed in Table 5. It could be seen that the weights of the five dimensions were perceived usefulness (0.102), perceived ease of use (0.069), perceived risk (0.313), trust (0.361), and satisfaction (0.154), respectively. It could be seen from the above weights that the order of degree of recognition of the five dimensions by the experts was trust, perceived risk, satisfaction, perceived usefulness, and perceived ease of use.

**Table 5:** Order of dimensions.

Ranking	Name of dimensions	Weight
1	Trust	0.361
2	Perceived risk	0.313
3	Satisfaction	0.154
4	Perceived usefulness	0.102
5	Perceived ease of use	0.069

### 3. SEM results

#### (1) Structural diagram on consumers and hypotheses

**Fig 4:**
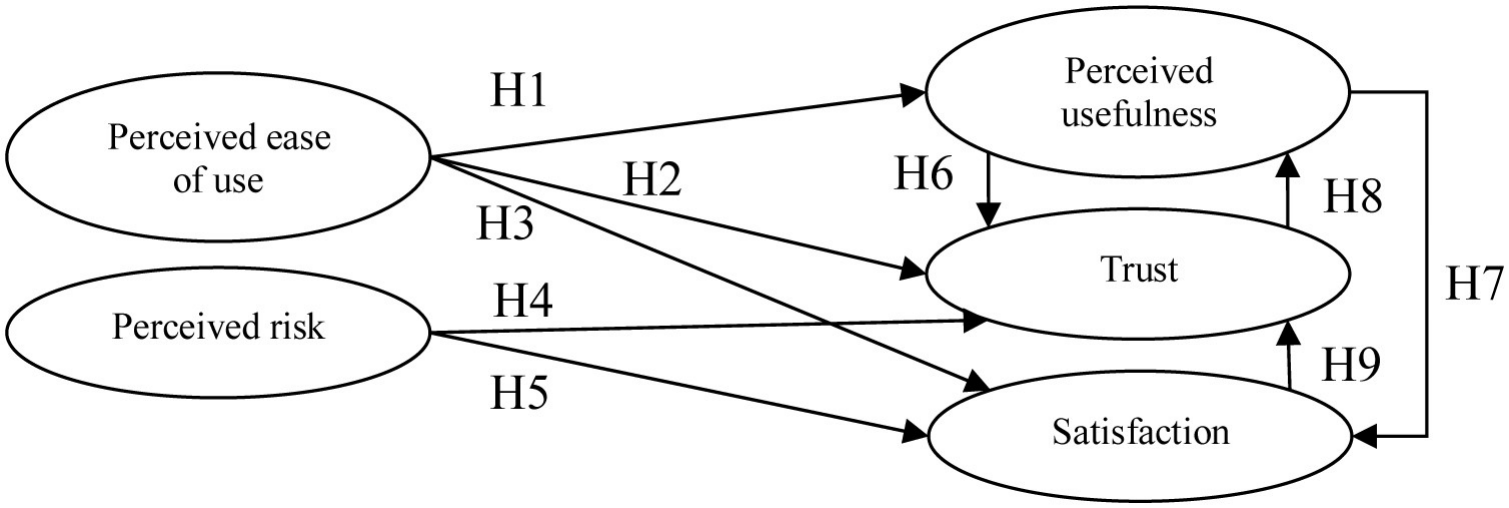
Structural relation diagram of dimensions.

In this study, based on the correlations among dimensions identified through the results of the DEMATEL questionnaires completed by experts, the architecture diagram from the aspect of consumer use was further designed, as shown in Fig 4, and the following hypotheses were proposed:

H1: Consumer perceived ease of use for Internet banking will influence the perceived usefulness.
H2: Consumer perceived ease of use for Internet banking will influence trust.
H3: Consumer perceived ease of use for Internet banking will influence satisfaction.
H4: Consumer perceived risk for Internet banking will influence trust.
H5: Consumer perceived risk for Internet banking will influence satisfaction.
H6: Consumer perceived usefulness for Internet banking will influence trust.
H7: Consumer perceived usefulness for Internet banking will influence satisfaction.
H8: Consumer trust in Internet banking will influence perceived usefulness.
H9: Consumer satisfaction with Internet banking will influence trust.

#### (2) Descriptive statistics

In order to identify the degree of recognition of each dimension by consumers, an online questionnaire survey was implemented in this study. A total of 398 questionnaires were recovered, including 373 valid questionnaires after removing 25 invalid questionnaires. The results of the descriptive statistics based on all valid questionnaires are listed in Table 6.

**Table 6:** Descriptive statistics of samples.

Sample characteristics		Number of people	Percentage (%)
Gender	Male	177	47.45%
	Female	196	52.55%
Age	18–20 years old	11	2.95%
	21–30 years old	67	17.96%
	31–40 years old	120	32.17%
	41–50 years old	114	30.56%
	51–60 years old	61	16.35%
Occupation	Manufacturing industry	32	8.58%
	Service industry	89	23.86%
	Finance and insurance	116	31.10%
	Information technology	61	16.35%
	Education	14	3.75%
	Student	30	8.04%
	Other industry	31	8.31%
Internet banking use experience	Less than one year	135	36.19%
	One to two years	106	28.42%
	Above two years	132	35.39%
Internet banking use frequency	At least one time per week	148	39.68%
	At least one time per month	135	36.19%
	At least one time per year	90	24.13%
Internet banking access	Desktop (Notebook) PC	142	38.07%
	Mobile device (smartphone, tablet PC)	231	61.93%

#### (3) Reliability and validity analysis

*1*. *Reliability*. Upon removing the invalid samples, it was necessary to confirm whether there was a change in the factor structure during the process of data deletion. Accordingly, prior to SEM verification, the reliability and validity were tested in this study to measure the goodness of fit of the model. The analysis results in respect of reliability are listed in Table 7. The Cronbach's α coefficients of all dimensions presented in the questionnaire were greater than 0.7, which demonstrated that the contents of the questionnaire designed in this study had a high internal consistency and that further data analysis could be continued.

**Table 7:** Reliability analysis of dimensions.

Name of dimension	Cronbach's α value
Perceived usefulness	0.869
Perceived ease of use	0.861
Perceived risk	0.734
Trust	0.890
Satisfaction	0.870

*2*. *Validity*. [81] believed that a factor loading greater than 0.5 is appropriate. Therefore, in this study, the construct validity of the research model was measured by factor analysis. As shown in Table 8, the factor loading values of all questions were greater than 0.5, which demonstrated that the model had high construct validity.

**Table 8:** Validity analysis (factor loading) of dimensions and item analysis.

Dimension	Item	Factor loading	Average mean	Dimension average
Perceived usefulness	(1) Internet banking improves my efficiency in conducting financial transactions.	0.88	4.20	4.05
	(2) Internet banking allows me to know about more banking businesses.	0.73	3.81	
	(3) Internet banking allows me to keep abreast of the latest banking services in a timely fashion.	0.63	3.99	
	(4) Internet banking allows me to use more banking services in a convenient manner.	0.88	4.21	
Perceived ease of use	(1) I think it is effortless to learn to use Internet banking.	0.83	4.21	4.24
	(2) In think it is effortless to be familiar with the operation of Internet banking.	0.75	4.14	
	(4) In think it is effortless to complete banking business via Internet banking.	0.85	4.29	
	(5) I think the interface process of Internet banking is clear and easy to understand.	0.70	4.31	
Perceived risk	(1) I think the security measures of Internet banking for transactions are inadequate.	0.60	4.09	4.13
	(2) In think Internet banking will be likely to leak consumers’ personal information to other institutions.	0.68	4.19	
	(3) I am afraid I will enter a wrong amount during online transactions.	0.57	4.13	
	(4) I think mistakes are likely to be made during transactions via Internet banking.	0.57	4.06	
	(5) I think Internet banking is likely to be subject to hacking, which will cause financial loss to consumers.	0.57	4.17	
Trust	(1) I think Internet banking should employ a set of optimized security mechanisms to improve the security of transaction data.	0.74	4.54	4.43
	(2) I think Internet banking should establish several backup systems to ensure normal use of Internet banking by consumers when the web is under attack.	0.89	4.60	
	(3) Internet banking will consider the interests and needs of users and provide the products and services they need.	0.90	4.57	
	(4) I think Internet banking should take precautionary measures for major events (such as malicious stealing of money).	0.71	4.34	
	(5) I think Internet banking should transfer the risk of potential loss due to major events to other vendors via insurance.	0.71	4.12	
Satisfaction	(1) Banks have a good image and reputation.	0.73	4.14	4.27
	(2) Internet banking can update the information on the web anytime.	0.79	4.10	
	(3) Internet banking has a stable and fast system.	0.76	4.31	
	(4) Customer service personnel have appropriate expertise and attitudes.	0.74	4.38	
	(5) Answers can be obtained regarding Internet banking in real time in case of any question.	0.76	4.41	

#### (4) Goodness of fit analysis

In this study, the goodness of fit of the model was further evaluated, as shown in Table 9. The Chi-Square value/DOF, RMSEA value, RMR value, and SRMR value fell within the acceptable range, which demonstrated that the model met the measurement index for the Overall Fix Index. In addition, the NFI, NNF, IFI, CFI, RFI, and PGFI values were greater than the acceptable range, which demonstrated that the model met the Comparative Fit Index and Parsimonious Fit Index.

**Table 9:** Goodness of fit indexes of the model.

Type	Name of index	Judged value	Measured value
Overall Fix Index	Chi-Square value/ DOF	<5	3.45
	RMSEA	<0.1	0.08
	RMR	<0.1	0.05
	SRMR	<0.1	0.05
Comparative Fit Index	NFI	>0.9	0.94
	NNFI	>0.9	0.95
	IFI	>0.9	0.96
	CFI	>0.9	0.96
	RFI	>0.9	0.93
Parsimonious Fit Index	PGFI	>0.5	0.67

#### (5) Verification of hypotheses

**Fig 5:**
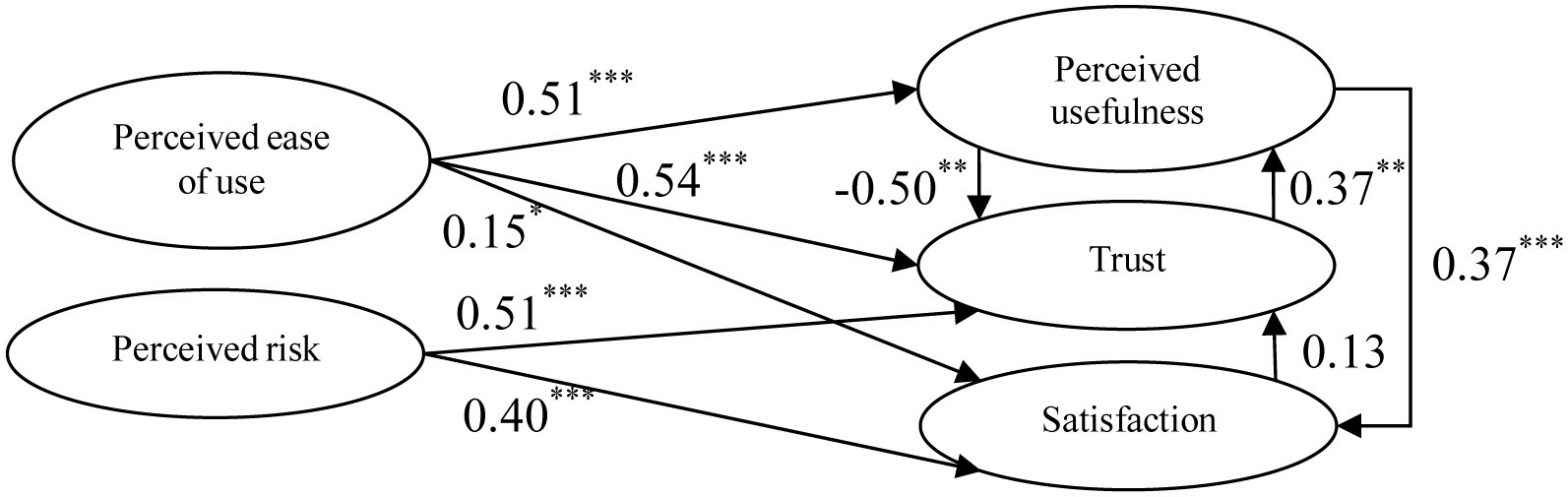
Diagram of research hypotheses.

Upon completion of verification of the reliability, validity, and goodness of fit of the model, nine research hypotheses for the model were verified in this study. A significant positive influence relationship was observed among all hypotheses except for H6 (significantly negative) and H9 (insignificant). The empirical results are shown in Table 10 and Fig 5. H9 failed, which demonstrated that consumers may not trust Internet banking even when it satisfies basic transaction requirements (such as deposits, foreign exchange, transfers, payment of fees, and other services), as there are still many risk factors to be considered (such as liquidity, information security, and compliance with financial laws and regulations). A significant positive correlation was found between H4: Consumer perceived risk for Internet banking will influence trust and H5: Consumer’s perceived risk for Internet banking will influence satisfaction, which was inconsistent with the result of prior literature. The reason behind this difference was that the dimension of perceived risk designed in this study was a reverse question; therefore, a lower occurrence of perceived risk represented higher trust and satisfaction. Internet banking has been developed in the Taiwan market for decades, and the system has been designed well; therefore, most consumers have gotten used to the benefits brought by the use of Internet banking. As a result, under the business model highlighting technological innovation and customer experience, consumers believe that industry operators will develop related coping strategies to address various risks, in order to allow them to feel safe and continue to use the services provided by Internet banking. Consumers may not trust the quality of services provided by Internet banking although they believe that Internet banking enables real-time operations and improves transaction efficiency, which demonstrated the result of H6. On the contrary, consumers believe that Internet banking will improve transaction speed and efficiency if they trust Internet banking more, which demonstrated the result of H8.

**Table 10:** Verification results of research hypotheses.

Description of hypotheses		Verification results	Path coefficient
H1:	Consumer perceived ease of use for Internet banking will influence perceived usefulness.	Supported	0.51***
H2:	Consumer perceived ease of use for Internet banking will influence trust.	Supported	0.54***
H3:	Consumer perceived ease of use for Internet banking will influence satisfaction.	Supported	0.15*
H4:	Consumer perceived risk for Internet banking will influence trust.	Supported	0.51***
H5:	Consumer perceived risk for Internet banking will influence satisfaction.	Supported	0.40***
H6:	Consumer perceived usefulness for Internet banking will influence trust.	Supported	-0.50**
H7:	Consumer perceived usefulness for Internet banking will influence satisfaction.	Supported	0.37***
H8:	Consumer trust with Internet banking will influence perceived usefulness.	Supported	0.37**
H9:	Consumer satisfaction with Internet banking will influence trust.	Not supported	0.13

*** refers to p<0.01,

** refers to p<0.05,

* refers to p<0.1.

#### (6) Item analysis in the aspect of consumer use

Upon completion of the verification of hypotheses, in this study, the degree of recognition of the five dimensions by consumers was further analyzed by analyzing the scales of the questionnaires completed by consumers, as shown in Table 11. It could be seen that among the five dimensions, the top three items recognized by consumers were still “I think Internet banking should establish several backup systems to ensure normal use of Internet banking by consumers when the web is under attack” (4.60), “Internet banking will consider the interests and needs of users and provide the products and services they need” (4.57), and “I think Internet banking should employ a set of optimized security mechanisms to improve the security of transaction data” (4.54). These three items all fell in with the dimension of trust. The degree of recognition of the five dimensions by consumers was ranked in a descending order: trust (4.43), satisfaction (4.27), perceived ease of use (4.24) perceived risk (4.13) and perceived usefulness (4.05).

**Table 11:** Comparison table of the degree of recognition of the five dimensions by experts and consumers.

Name of dimension	Expert	Consumer
Perceived usefulness	4	5
Perceived ease of use	5	3
Perceived risk	2	4
Trust	1	1
Satisfaction	3	2

#### (7) Comparisons of the degree of recognition of the five dimensions by experts and consumers

A comparison table of the degree of recognition of the five dimensions by experts and consumers was prepared based on Tables 5 and 8. As listed in the table, the dimension of trust was prioritized by both experts and consumers, which demonstrated that experts and consumers believe that Internet banking should develop a set of optimized security mechanisms to safeguard the security of data and websites, as well as to prevent any loss to users’ assets by prioritizing the interests and requirements of users. However, except for the same view of experts and consumers on the dimension of trust, different views were observed on other dimensions. Consumers put more emphasis on the dimension of satisfaction, while experts put more emphasis on the dimension of perceived risk, which demonstrated that consumers attach more importance to the assistance of customer service personnel obtained in real time during the online services provided by Internet banking, while experts attach more importance to risk control and reducing people’s perceived risk for Internet banking.

Through comparisons of the degree of recognition of the five dimensions by experts and consumers, the business strategies for Internet banking of banking directors and the actual use by consumers were identified. Banking industry operators may make a reference to the opinions of both experts and consumers and make a cross analysis in developing business decisions on Internet banking in the future, in order to obtain the best business decision. Consumers may also select the optimal Internet banking based on the research results in using Internet banking.

## V. Conclusion

Internet banking is a financial service that has been developed by banking operators for many years, and consumers have already gotten used to the services provided by Internet banking. With the rise of financial technology, however, consumers have access to financial services from more diversified channels other than traditional institutions. Therefore, Internet banking is faced with increasingly fierce competition. Most prior studies discussing Internet banking did not analyze or compare aspects of the business operator and consumer use at the same time. In view of the above, the business strategies for Internet banking of banking directors and the actual use by consumers were identified through expert and consumer questionnaires, respectively, and DEMATEL, ANP and SEM techniques were applied to analyze the degree of recognition of the factors influencing the use of Internet banking by experts and consumers. The research findings were as follows:

### 1. Business strategies for Internet banking of banking directors

**Fig 6:**
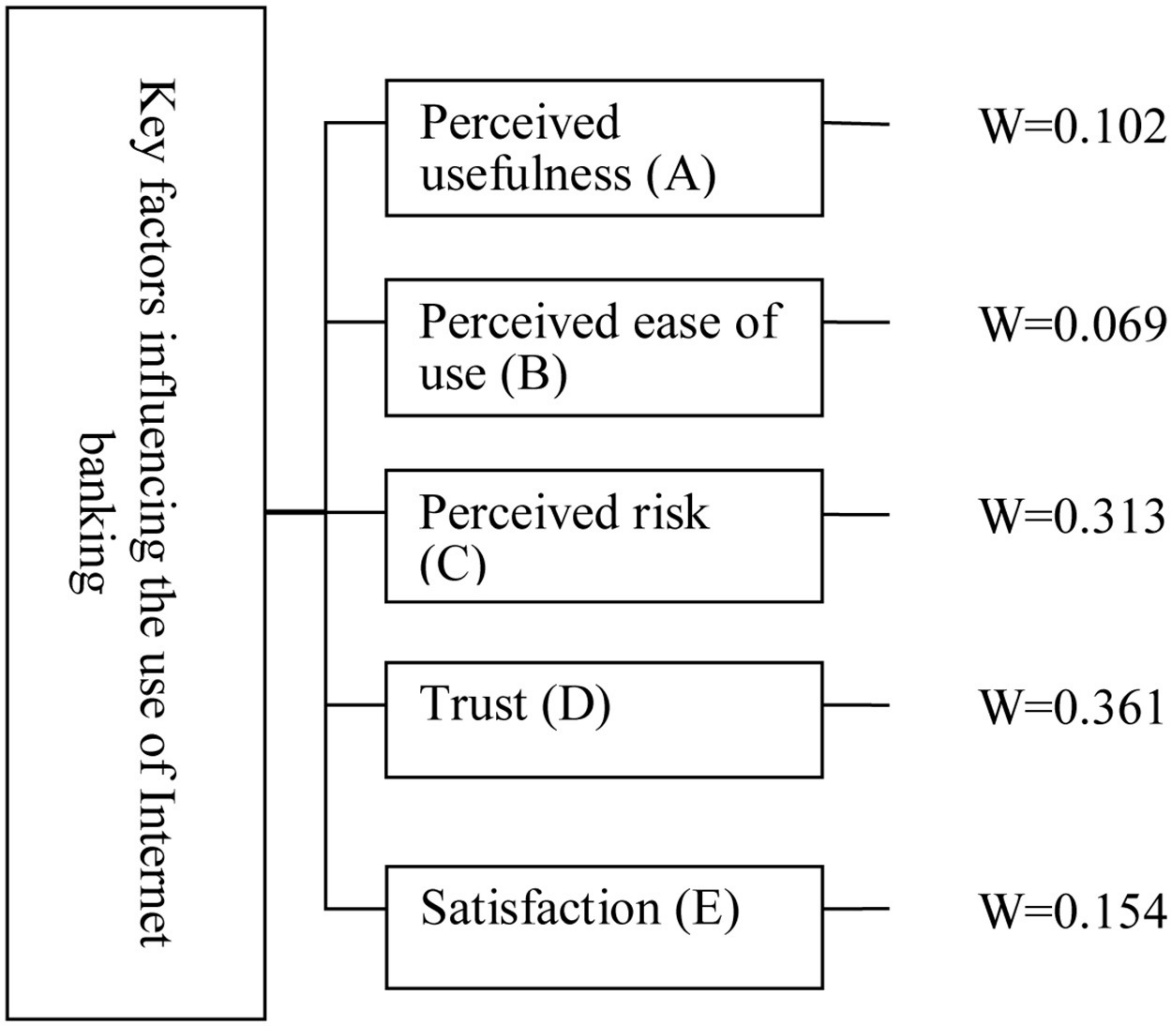
Weight of factors.

In this study, the five factors influencing the use of Internet banking were identified through a literature review. First, the architecture in the aspect of the operator established in this study was evaluated by the DEMATEL technique. The empirical results showed influence and certain correlations among the five factors, as shown in Table 12. Second, the weights of the factors were calculated by the ANP technique and Super Decision software to further identify the key factors influencing the use of Internet banking, as shown in Fig 6.

**Table 12:** Correlations among factors.

Factor	Influence on other factors	Subject to influence of other factors
Perceived usefulness (A)	D, E	B
Perceived ease of use (B)	A, D, E	Nil
Perceived risk (C)	D, E	Nil
Trust (D)	A	A, B, C, E
Satisfaction (E)	D	A, B, C

The empirical results showed that banking directors consider the factor of trust as the most important influence factor, which demonstrated that the experts’ business strategies for Internet banking focus on data maintenance and system security. Accordingly, this study believed that banking industry operators should develop optimized security protection mechanisms and simulate and develop a set of standard operating procedures for potential malicious attack incidents in the future, so as to improve banks’ crisis management abilities and reduce consumer concerns about the security of Internet banking.

### 2. Actual use of internet banking by consumers

Correlations among the five influence factors were discussed by the DEMATEL technique in this study, after which research hypotheses were proposed and the architecture in the aspect of consumer use was developed and verified by SEM. The degree of recognition of the five factors by consumers was identified by a questionnaire survey, as shown in Table 13.

**Table 13:** Degree of recognition of the five factors by consumers.

Factor	Score (1–5 scores)	Ranking
Perceived usefulness	4.05	5
Perceived ease of use	4.24	3
Perceived risk	4.13	4
Trust	4.43	1
Satisfaction	4.27	2

As shown in Table 13, consumers consider the factor of trust as the most important influence factor. That is to say, when using Internet banking, consumers will consider whether the security protection of Internet banking is acceptable and whether the products and services provided by the banking operators are actually needed. As a result, this study believed that banking operators should develop an optimized security protection mechanism and establish a system in Internet banking to evaluate the risk preference of investors and provide the services required by customers.

### 3. Comparisons of views of banking directors and consumers

The empirical results showed that the factor of trust is considered as the most important influence factor by both banking operators and consumers, while the degree of recognition of other influence factors varies. Accordingly, the views of both parties were integrated and the following suggestions were proposed:

1. Banking operators should improve the security mechanism of Internet banking and develop several backup systems to ensure safe use. In addition, banking operators should consider the interests and needs of consumers to provide the products and services they need, in order to improve consumer’s trust with Internet banking.
2. Banking operators should cultivate the expertise and quality of customer service personnel of Internet banking, in order to ensure that consumer concerns will be addressed in real time, and further increase consumer satisfaction with Internet banking.
3. Banking operators should design an easy-to-operate interface according to the actual use by consumers, in order to enable consumers to address difficulties through self-learning in terms of operation and make them have faith in completing banking business via Internet banking.
4. Banking operators should inform consumers of the latest services and preferential measures regularly, in order to make them believe that using Internet banking will bring the benefits and improve the execution efficiency of financial transactions.

### 4. Research limitations and suggestions

The limitation of this research mainly lies in the data collection. Expert interviews were conducted for data collection and analysis in the first stage of this research. Although the interviews aimed for objectivity, but biases might still occur due to the subjective consciousness of the respondents. In this regard, it is suggested that the number of respondents may be increased in the future research to make their judgment closer to the actual situations. In addition, the research hypothesis was established based on the results of DEMATEL, which was completely different from the traditional models. After the establishment of the hypothesis, it was necessary to discuss its rationality with the respondents of the first stage, making the entire process time-consuming. With more researches on online banking in the future, researchers may confirm the hypothesis rationality of this study.

## VI. How to popularize and management implications

With the advancement of the technology of big data analysis, online banking can recommend commodities more accurately after the user behavior analysis, and facilitate online trades, gradually leading to an increase in online business revenue. Banks may establish event-based marketing mechanisms to identify the users’ needs based on the changes in their behaviors, and then actively embed proper advertisements. For example, if the customers are found to have never purchased any fund in the past but to frequently browse the fund-related information in recent weeks, this indicates that the customers might be interested in fund investments, so the preferential activities of the current funds and selected fund information may be actively offered. For another example, customers often spend one to two months finding their next investment target, during the meantime, the bank may also actively recommend products suitable to the customer according to their risk attributes. Nowadays, banks not just unilaterally recommend products but emphasize interaction on websites and customer feedback, which indicates that website operation transforms from function orientation to customer demand orientation. From the perspective of overall benefits, establishing online banking can reduce service costs and increase commission incomes, as well as enhance brand images and increase customer loyalty.

Online banking is an essential and important service in the development of banking industry. From the perspective of bankers, transaction with online banking can provide customers with more diversified financial services, and reduce the internal labor costs, so as to reduce operating costs and improve service efficiencies. From the perspective of customers, online banking not only provides 24-hour transaction services, but also enables customers to easily access the previous transaction data. In recent years, the integrated management services of personal financial commodities have been developed. Customers can benefit from reduced or waived online banking fees, such as transfer and foreign exchange. Hence, the operation of online banking can create a win-win situation for both banks and customers. In recent years, the ratio of electronic transaction has risen and e-commerce platforms have become one of the representative images of financial institutions. Customers attach great importance to the stability of online banking systems, the ease of use of operating interfaces, the security of transaction data, and the speed of information transmission, so the improvement of service quality and system quality is also an issue that online banking service providers must pay attention to.

In the future, financial institutions should focus on developing online banking systems that are personalized, convenient to use, and consistent with the consumption trend. On the other hand, online banking systems with high stability, efficiency, and security are easier to be developed and maintained, and have flexibility, which can save the future development and maintenance costs. Practically, financial and marketing functions are added in cooperation with business planning to develop innovative banking business, to further integrate financial services with personal and corporate needs, and to make all kinds of financial commodity information more popular in a faster and timelier manner. Financial institutions no longer only consider establishing more branches to expand their business, but re-examine the cost structures and operating expenses, as well as effectively use information technology, so as to change their traditional business models and the previous competition patterns of banks. Online banking is not only about the transactions between banks and customers, but also provides platforms for bankers to understand the information and services needed by customers, so as to make financial services better meeting customers’ needs.
